# Open ventral hernia repair with a composite ventral patch - final results of a multicenter prospective study

**DOI:** 10.1186/s12893-019-0555-z

**Published:** 2019-07-16

**Authors:** F. Berrevoet, C. Doerhoff, F. Muysoms, S. Hopson, M. G. Muzi, S. Nienhuijs, E. Kullman, T. Tollens, M. Schwartz, K. Leblanc, V. Velanovich, L. N. Jørgensen

**Affiliations:** 1 Department of General and Hepatopancreatobiliary Surgery, Ghent University Hospital, Corneel Heymanslaan 10, 9000 Ghent, Belgium; 2 Surgicare of Missouri, Jefferson City, MO USA; 3 Department of Surgery AZ Maria Middelares, Ghent, Belgium; 4 Bon Secours Hernia Center, Mary Immaculate Hospital, Newport News, VA USA; 5 University Hospital Tor Vergata, Rome, Italy; 6 Department of Surgery, Catharina Hospital, Eindhoven, Netherlands; 7 HELSA Specialist Center, Linköping, Sweden; 8 Imelda Hospital -General Surgery Imelda Hospital, Bonheiden, Belgium; 9 Monmouth Medical Center, Long Branch, NJ USA; 10 Our Lady of Lakes Regional Medical Center, Baton Rouge, LA USA; 11 Division of General Surgery, University of South Florida, Tampa General Hospital, Tampa, FL USA; 12 Digestive Disease Center, Bispebjerg Hospital, University of Copenhagen, Copenhagen, Denmark

**Keywords:** Ventral hernia, Epigastric hernia, Umbilical hernia, Intraperitoneal mesh, Surgical mesh, Parietex™ composite ventral patch

## Abstract

**Background:**

This study assessed clinical outcomes, including safety and recurrence, from the two-year follow-up of patients who underwent open ventral primary hernia repair with the use of the Parietex™ Composite Ventral Patch (PCO-VP).

**Methods:**

A prospective single-arm, multicenter study of 126 patients undergoing open ventral hernia repair for umbilical and epigastric hernias with the PCO-VP was performed.

**Results:**

One hundred twenty-six subjects (110 with umbilical hernia and 16 with epigastric hernia) with a mean hernia diameter of 1.8 cm (0.4–4.0) were treated with PCO-VP. One hundred subjects completed the two-year study. Cumulative hernia recurrence was 3.0% (3/101; 95%CI: 0.0–6.3%) within 24 months. Median Numeric Rating Scale pain scores improved from 2 [0–10] at baseline to 0 [0–3] at 1 month (*P* < 0.001) and remained low at 24 months 0 [0–6] (*P* < 0.001). 99% (102/103) of the patients were satisfied with their repair at 24 months postoperative.

**Conclusions:**

The use of PCO-VP to repair primary umbilical and epigastric defects yielded a low recurrence rate, low postoperative and chronic pain, and high satisfaction ratings, confirming that PCO-VP is effective for small ventral hernia repair in the two-year term after implantation.

**Trial registration:**

The study was registered publically at clinicaltrials.gov (NCT01848184 registered May 7, 2013).

## Background

Although there is no consensus on the ideal technique for repairing umbilical or epigastric hernias in adults, to our knowledge only two prospective randomized trials have been conducted. The first by Arroyo et al. showed an 11% versus 1% recurrence rate after primary suture and mesh repair [1]. While the second, by Polat et al. compared 3 different techniques in umbilical hernia repairs (Prolene Hernia System, Mayo repair, and onlay repair with mesh) and demonstrated recurrence rates of 0, 11, and 0%, respectively [2]. According to Arroyo et al. and also retrospectively shown by Sanjay et al., the conclusion could be drawn that the use of mesh in these patients is mandatory, no matter the diameter of the defect [3]. Due to the technical difficulty of retrorectus and preperitoneal dissection for these small hernias, an alternative approach is to use a self expanding mesh device that can be introduced via an incision at the level of the hernia into the peritioneal cavity. Once deployed, traction on the fixation points or straps leads to a flat alignment to the abdominal wall. As a quick and elegant procedure, these devices have been embraced by many surgeons.

However, further studies on the two most frequently used mesh devices revealed several issues influencing clinical outcomes. Cases of serious complications due to severe adhesion formation as well as higher recurrence rates compared to traditional retromuscular mesh placement have been reported [4–6]. Thus, in addition to the design of the mesh device, adequate handling of the fatty intra-abdominal structures (both falciform ligament and umbilical folds) is mandatory to achieve best outcomes. The currently available devices for repair of small ventral hernias consist of polypropylene (PP) in combination with well known anti-adhesive barriers, as ePTFE, sepramesh technology or oxidized cellulose.

Several earlier reports also mention issues with deployment using a two-strap fixation method [4, 7]. Instead, by using a four-flap fixation, flat alignment to the abdominal wall might be improved. This manuscript reports the final two-year clinical results of a prospective study of patients undergoing open ventral hernia repair with intra-peritoneal positioning of a recently developed patch for hernia repair.

## Methods

As reported in the early results of this prospective multicenter cohort study, patients underwent a small primary ventral hernia repair using the Parietex™ Composite Ventral Patch (PCO-VP) (Medtronic; Trevoux, France) with a diameter of 4.6, 6.6, or 8.6 cm, depending on the size of the defect [8]. The objectives were to assess hernia recurrence at 24 months follow-up and safety. All patients ≥18 years of age planned for primary ventral hernia surgery via open approach were consented if they met eligibility criteria via a screening/baseline visit within 6 weeks of their procedure. All in- and exclusion criteria were reported previously with patients in need of emergency surgery, pregnant patients and BMI > 35 kg/m^2^ being excluded. The surgical procedure applied was described in detail earlier [9]. In short, opening of the hernia sac was performed, and the fascial defect was measured. The sac was dissected out and opened and possible contents were reduced. A finger was then inserted into the defect to clear the surrounding peritoneum. The PCO-VP was then inserted into the peritoneal cavity. After introduction, the mesh is pulled up gently, to flatten the patch against the abdominal wall. To fix the mesh against the abdominal wall the 4 quadrants of the patch were fixed to the margins of the fascia defect with 4 non-resorbable sutures. Although not specifically recommended, the anterior fascia was then closed over the mesh to minimize the risks for mesh infection. For hernias < 1 cm in diameter any size of PCO-VP could be used. For hernias 1–3 cm in diameter, 6.6 and 8.6 cm PCO-VP were recommended, and for hernias > 3 cm in diameter, 8.6 cm PCO-VP was recommended. The meshes were deployed in the intraperitoneal position. If intraperitoneal positioning was difficult, preperitoneal placement was performed. Laparoscopic control was only performed in 10 patients as described earlier [8], all other patients had no laparoscopy done at the end of the procedure.

The primary endpoint was recurrence at 24 months evaluated by a physical examination and by ultrasonography in all patients (not only in case of suspicion for recurrence). The secondary endpoints were recurrence at one, six- and 12-months post-surgery. Additional outcomes included: postoperative pain measured by the Numeric Rating Scale (NRS) 0–10 in accordance with the definition in 4 categories (0 = No pain, 1–3 = minor/mild pain, 4–6 = moderate pain, 7–9 = severe pain, and 10 = worst pain); postoperative patient comfort measured by the Carolina’s Comfort Scale® (CCS) to assess quality of life, as well as adverse events (AEs).

### Statistics

As the recurrence rate for primary ventral hernia repair using synthetic mesh is approximately 4% (range: 0–6%) [5, 7–10], assuming a 95% confidence interval, 60 patients were needed for the evaluable population. Anticipating a 20% lost to follow-up rate a minimum of 100 patients were required as the evaluable population. Recurrence rates and complications were analyzed using time to event (Kaplan-Meier) analysis, while Student t-test or non-parametric Mann-Whitney test were used for mean comparisons between sub-groups. Proportion comparisons between sub-groups were performed using Chi-square or Fisher’s exact test. All tests were performed using two-side tests with an α-level of 5%. Analyses were performed using SAS® Version 9.2 or higher (SAS Inc., Cary, NC).

This study was conducted in accordance with the Declaration of Helsinki (2008), ICH–GCP guidelines, ISO 14155-2011, institutional review board, and ethics council approval. The study was registered publically at clinicaltrials.gov (NCT01848184).

## Results

One hundred twenty-six patients, including 87 males and 39 females, were enrolled between May 3, 2013 and July 12, 2016 and treated by 12 surgeons in 12 different centers in Europe and the United States. A total of 110 (87.3%) patients were assessed at 12 months and 100 (79.4%) patients were assessed at 24 months for hernia recurrence and completed the two-year clinical assessment for the primary endpoint. Three additional patients were assessed by phone at the 24-month follow-up. Most patients who exited the study before 24 months were lost to follow-up (9.5%, *n* = 12/126), withdrew voluntarily (6.3%, *n* = 8/126), were unable or unwilling to participate in follow-up visits (2.4%, *n* = 3/126), exited due to AEs (1.6%, *n* = 2/126), or death (0.8%, *n* = 1/126) that was not procedure or device related. Most patients (*n* = 110) were treated for an umbilical hernia and the remainder (*n* = 16) were treated for epigastric hernia. Forty-three (34.1%, *n* = 43/126) patients had a BMI of at least 30 kg/m^2^, and 90 (71.4%, *n* = 90/126) patients had at least one risk factor at baseline, including smoking (42.9%, *n* = 54/126), type II diabetes (7.9%, *n* = 10/126), and chronic obstructive pulmonary disease (7.1%, *n* = 9/126). The median operative time was 33 min [range, 10–93 min].

In total, 3 hernia recurrences (3.0%, *n* = 3/101); 95%CI: 0.0–6.3%) occurred within 2 years of repair (79 days, 197 days, and 288 days after surgery) including one patient who withdrew from the study after recurrence at 79 days. Reoperations were performed for two patients: A 33-year old female with BMI of 22.6 kg/m^2^ who had received an 8.6 cm PCO-VP for a 2.2 cm umbilical hernia repair experienced a hernia recurrence 79 days after surgery. A physical exam and ultrasound confirmed the recurrence. A laparoscopic reoperation confirmed a probable missed second hernia defect cephalad to the PCO-VP. Extensive adhesiolysis from omentum and colon was necessary to identify the defect and the PCO-VP prosthesis was removed. A 15 × 19 cm piece of mesh was placed intraperitoneally. The second patient was a 54-year old male with BMI of 28.4 kg/m^2^ who underwent umbilical hernia repair for a 3 cm hernia with an 8.6 cm PCO-VP. This subject experienced a hernia recurrence 197 days after surgery and eventually underwent laparoscopy demonstrating 50% cupping of the PCO-VP causing the recurrence, and an open preperitoneal repair with a 9 × 16 cm mesh was performed leaving the PCO-VP in place. The third patient with recurrence was a 69-year old female patient with a BMI of 34.7 kg/m^2^ who had a recurrence at 288 days. Her 1.2 cm umbilical hernia had been repaired by preperitoneal mesh placement using a 4.6 cm PCO-VP. During the original repair, the surgeon had difficulty with the intraperitoneal dissection and the mesh was placed preperitoneally rather than intraperitoneally mainly due to the patient’s obesity. Reoperation for the recurrence was not performed as it was asymptomatic.

Throughout the two-year study, 51 (40.5%, *n* = 51/126) subjects experienced at least 1 single AE, including 26 incidents (32.1%) of device-related AE, 54 incidents (66.7%) of procedure-related AE, and a total of 30 incidents (37%) of serious AE (SAEs). Twelve patients (9.5%, *n* = 12/126) had 2 AEs, 6 (4.8%, *n* = 6/126) patients had 3 AEs, and 2 (1.6%, *n* = 2/126) patients had 4 AEs. Table 1 describes the number of patients who reported procedure or device related AEs.

**Table 1:** Summary of incidence of specified AEs from baseline through 24-month follow-up

	*N* = 126
Subject with at least 1 AE	51 (40.5%)
Pain	27 (21.4%)
Abdominal pain due to recurrence	1 (0.8%)
Wound infection	5 (4.0%)
Hernia recurrence	3 (2.4%)
Haematoma at incision site	2 (1.6%)
Seroma	2 (1.6%)
Other^a^	5 (4.0%)

Data are presented in number of subjects, n (%). ^a^Other includes anemia 1(0.8%), occasional feeling of tightness at the operative site 1(0.8%), serious drainage at incision site 1(0.8%), Skin irritation 1(0.8%), and puncture of small bowel 1(0.8%)

**Fig. 1:**
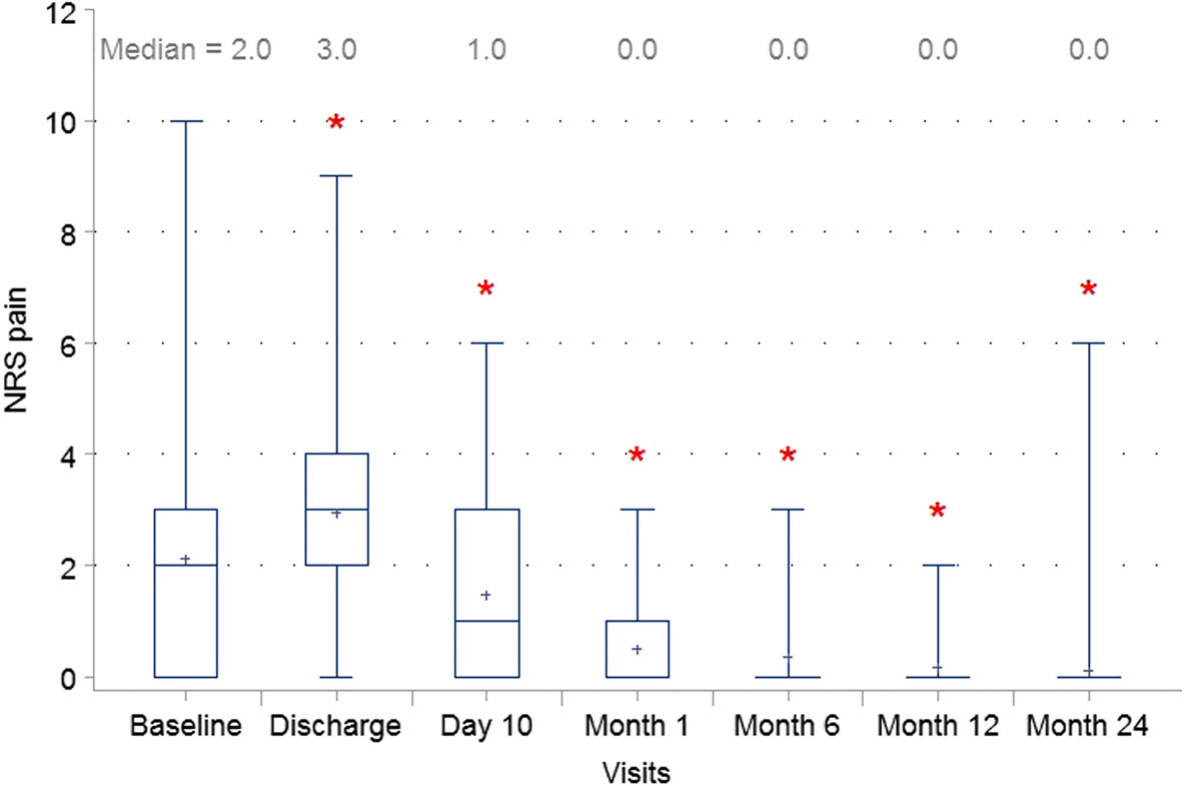
Numerical Rating Scale Pain Assessment. Patient pain levels by post-operative visit. Whiskers are drawn from quartiles (Q1 – Median – Q3 to the extreme values of the group) * indicates *P* < 0.05 relative to baseline

**Fig. 2:**
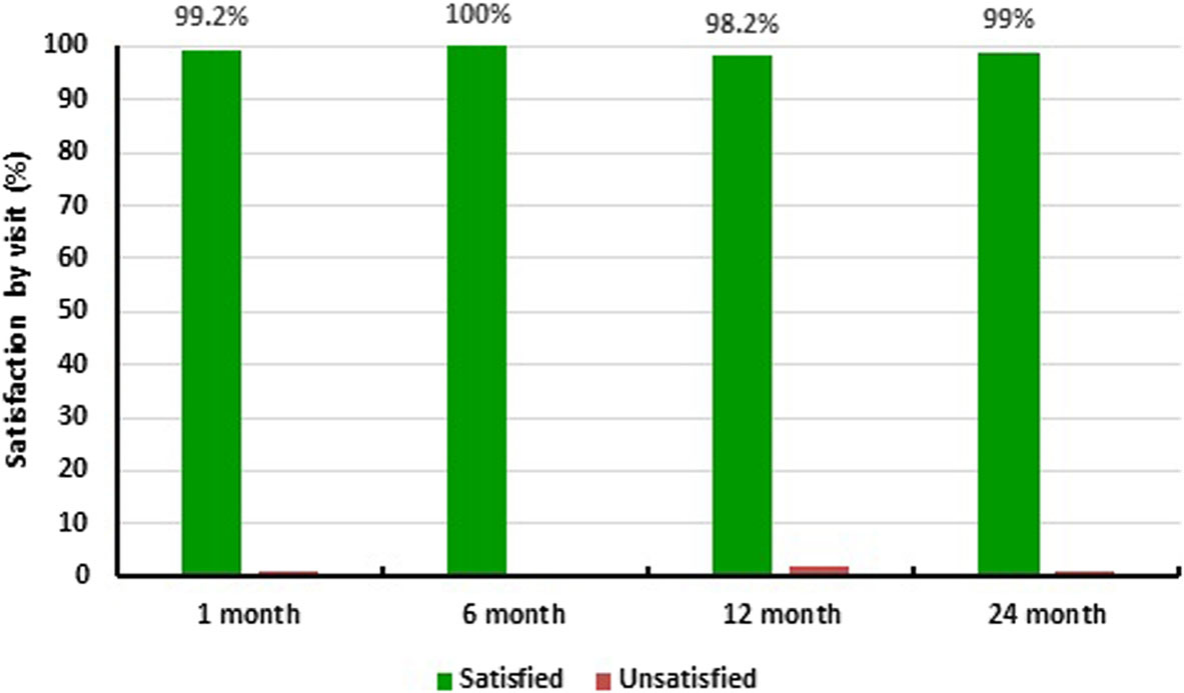
Patient satisfaction by percentage at visits 1-, 6-, 12- and 24-months postoperative

Postoperative numeric rating scale (NRS) pain scores are depicted in Fig. 1. Scores improved from a median [min-max] of 2 [0–10] at baseline to 0 at 24 months [0–6]. At baseline 34/120 (28.3%) patients experienced no pain. These values improved to 77/123 (62.6%) at 1 month and 96/103 (93.2%) at 24 months. Global Carolina’s™ Comfort Scale score improved from 1 [0–36] at 1 month postoperative to 0 [0–39] (*P* < 0.001) at 2 years post-surgery. When patients were asked to rate their satisfaction with the outcome of their procedure, the vast majority of patients responded as “completely satisfied” at all points of assessment, and 99% (102/103) of patients were satisfied with their repair at 24 months postoperative (Fig. 2). One patient responded as “unsatisfied” at his last assessment—he was the patient with hernia recurrence at 197 days.

## Discussion

Until now the use of different mesh devices has led to unacceptable morbidity, including gastrointestinal obstructions, mesh erosions, as well as high recurrence rates compared to other types of repair. In the present study more than 100 patients with umbilical hernia and epigastric hernia repair using the PCO-VP were investigated over the course of 2 years. Historically, these types of hernias, with relatively small defect diameters between 0.5 cm and 3 cm, have been primarily repaired using either suture closures, or the Mayo technique, yielding recurrence rates of 10–30% [10, 11]. As evident from this and similar studies, the use of a prosthetic mesh significantly reduces the mid-term 2 year rates of hernia recurrence even for small defects [1, 12, 13]. Recurrence rates of up to 2.2% (range, 1–2.2%) have previously been demonstrated using the mesh repair [1, 13] and similarly our study yielded a cumulative hernia recurrence rate of 3.0% within 24 months. With PCO-VP, the most common AEs reported were pain between discharge and 1-month follow-up (*n* = 27, 21.4%), and superficial wound dehiscence (*n* = 5, 4.0%). Pre-existing risk factors did not predispose subjects to AEs, although obesity might complicate mesh introduction in the intraperitoneal position as observed in 1 patient, which was later complicated by a recurrence.

The cupping phenomenon or “potato-chip” deformities are well-known issues after the use of mesh devices for small ventral hernia repair. These complications are probably due to excessive traction on the straps in order to align the patch flatly against the parietal peritoneum [4, 7]. Although mesh misalignment may also have occurred in this series [8], as laparoscopic evaluation is the only way to verify this, the low recurrence (3%) and reoperation rate observed at the end of the 2 year follow up, show that at least the clinical impact of misalignment has been minimal. Nevertheless, accurate surgical technique with complete dissection of the falciform ligament cranially and the umbilical folds caudally is mandatory to obtain best patient outcomes, especially when using the largest 8.6 cm patch. Garcia-Moreno et al. specifically examined the prothetic design of PCO-VP, in which the authors concluded, that both the intraperitoneal positioning and the incorporation of the mesh in the abdominal wall were very reliable using the four flap handles and fixation [14]. The well-known anti-adhesive properties of its collagen barrier to intraperitoneal adhesions have already proven its efficacy [15–17].

Our observed recurrence rate of 3.0% is lower than that previously reported with other mesh devices (up to 14.8% in 25 months [4, 13, 18–21], up to 12% at 16 months follow-up [22–26], or up to 10% after 43 months according to the regional cohort study from the Danish Hernia Database [27]). It is remarkable that a follow-up of 2 years in our studied patients did not increase the number of recurrences, despite a final ultrasound evaluation in all patients. However, it should be noted that this study does not exclude the possibility of later recurrences and that the actual recurrence rate might be slightly higher due to the number of lost to follow-up patients. In this respect, a randomized controlled trial comparing 2 different mesh devices could reinforce the value of these results.

As reported previously, the early wound morbidity was low (4.0% incidence of superficial wound dehiscence) without any evidence of mesh infection. But, as the study is limited by the absence of a control group, no further additional conclusions about the PCO-VP compared to alternate devices or surgical mesh repair techniques can be drawn. PCO-VP provides a potentially better outcome than reported by Bontinck et al., who reported 3% (3/96) of their patients had moderate or severe pain 12 months postoperative [23]; this difference might possibly be explained by their recurrence rate and less inflammatory and foreign body reaction after implantation of the PCO-VP [14]. In our study the excellent patient outcome is reflected in the quality of life global CCS score, which incorporates the sensation, pain and movement scores.

## Conclusion

The use of PCO-VP for the repair of primary umbilical and epigastric defects yielded a low recurrence rate, low postoperative and chronic pain, and high satisfaction ratings, confirming that PCO-VP is effective for small ventral hernia repair in the two-year term after implantation.

## Data Availability

The datasets generated and analysed during the current study are not publicly available due to restrictions that apply to the availability of these data, which were used under license for the current study, and so are not publicly available. Data are however available from the authors upon reasonable request and with permission of Medtronic.

## Abbreviations

AE: Adverse event
BMI: Body mass index
CCS: Carolina’s Comfort Scale®
NRS: Numeric rating scale
PCO-VP: Parietex™ Composite Ventral Patch
PP: Polypropylene

## References

[1] Arroyo A, Garcia P, Perez F, Andreu J, Candela F, Calpena R (2001). Randomized clinical trial comparing suture and mesh repair of umbilical hernia in adults. Br J Surg.

[2] Polat C, Dervisoglu A, Senyurek G, Bilgin M, Erzurumlu K, Ozkan K (2005). Umbilical hernia repair with the prolene hernia system. Am J Surg.

[3] Sanjay P, Reid TD, Davies EL, Arumugam PJ, Woodward A (2005). Retrospective comparison of mesh and sutured repair for adult umbilical hernias. Hernia.

[4] Berrevoet F, Van den Bossche B, de Baerdemaeker L, de Hemptinne B (2010). Laparoscopic evaluation shows deficiencies in memory ring deployment during small ventral hernia repair. World J Surg.

[5] Moreno-Egea A, Carrillo-Alcaraz A, Soria-Aledo V (2013). Randomized clinical trial of laparoscopic hernia repair comparing titanium-coated lightweight mesh and medium-weight composite mesh. Surg Endosc.

[6] Keating JJ, Kennedy GT, Datta J, Schuricht A (2016). Outcomes of 157 V-patch(TM) implants in the repair of umbilical, epigastric, and incisional hernias. Am Surg.

[7] Martin DF, Williams RF, Mulrooney T, Voeller GR (2008). Ventralex mesh in umbilical/epigastric hernia repairs: clinical outcomes and complications. Hernia.

[8] Berrevoet F, Doerhoff C, Muysoms F, Hopson S, Muzi MG, Nienhuijs S (2017). A multicenter prospective study of patients undergoing open ventral hernia repair with intraperitoneal positioning using the monofilament polyester composite ventral patch: interim results of the PANACEA study. Med Devices (Auckl).

[9] Berrevoet F, D'Hont F, Rogiers X, Troisi R, de Hemptinne B (2011). Open intraperitoneal versus retromuscular mesh repair for umbilical hernias less than 3 cm diameter. Am J Surg.

[10] Nardi MJ, Millo P, Brachet Contul R, Fabozzi M, Persico F, Roveroni M (2012). Laparoscopic incisional and ventral hernia repair (LIVHR) with PARIETEX composite mesh. Minim Invasive Ther Allied Technol.

[11] Halm JA, Heisterkamp J, Veen HF, Weidema WF (2005). Long-term follow-up after umbilical hernia repair: are there risk factors for recurrence after simple and mesh repair. Hernia.

[12] Aslani N, Brown CJ (2010). Does mesh offer an advantage over tissue in the open repair of umbilical hernias? A systematic review and meta-analysis. Hernia.

[13] Christoffersen MW, Helgstrand F, Rosenberg J, Kehlet H, Bisgaard T (2013). Lower reoperation rate for recurrence after mesh versus sutured elective repair in small umbilical and epigastric hernias. A nationwide register study. World J Surg.

[14] Garcia-Moreno F, Perez-Lopez P, Sotomayor S, Perez-Kohler B, Bayon Y, Pascual G (2015). Comparing the host tissue response and peritoneal behavior of composite meshes used for ventral hernia repair. J Surg Res.

[15] Balique JG, Benchetrit S, Bouillot JL, Flament JB, Gouillat C, Jarsaillon P (2005). Intraperitoneal treatment of incisional and umbilical hernias using an innovative composite mesh: four-year results of a prospective multicenter clinical trial. Hernia.

[16] Deeken CR, Faucher KM, Matthews BD (2012). A review of the composition, characteristics, and effectiveness of barrier mesh prostheses utilized for laparoscopic ventral hernia repair. Surg Endosc.

[17] Chelala E, Barake H, Estievenart J, Dessily M, Charara F, Alle JL (2016). Long-term outcomes of 1326 laparoscopic incisional and ventral hernia repair with the routine suturing concept: a single institution experience. Hernia.

[18] Ammaturo C, Bassi UA, Bassi G (2010). Outcomes of the open mesh repair of large incisional hernias using an intraperitoneal composite mesh: our experience with 100 cases. Updat Surg.

[19] Iversen E, Lykke A, Hensler M, Jorgensen LN (2010). Abdominal wall hernia repair with a composite ePTFE/polypropylene mesh: clinical outcome and quality of life in 152 patients. Hernia.

[20] Tollens T, Den Hondt M, Devroe K, Terry C, Speybroeck S, Aelvoet C (2011). Retrospective analysis of umbilical, epigastric, and small incisional hernia repair using the Ventralex hernia patch. Hernia.

[21] Vychnevskaia K, Mucci-Hennekinne S, Casa C, Brachet D, Meunier K, Briennon X (2010). Intraperitoneal mesh repair of small ventral abdominal wall hernias with a Ventralex hernia patch. Dig Surg.

[22] Ambe P, Meyer A, Kohler L (2013). Repair of small and medium size ventral hernias with a proceed ventral patch: a single center retrospective analysis. Surg Today.

[23] Bontinck J, Kyle-Leinhase I, Pletinckx P, Vergucht V, Beckers R, Muysoms F (2014). Single centre observational study to evaluate the safety and efficacy of the proceed ventral patch to repair small ventral hernias. Hernia.

[24] Muysoms FE, Bontinck J, Pletinckx P (2011). Complications of mesh devices for intraperitoneal umbilical hernia repair: a word of caution. Hernia.

[25] Tollens T, Struyve D, Aelvoet C, Vanrijkel JP (2010). Introducing the proceed ventral patch as a new device in surgical management of umbilical and small ventral hernias: preliminary results. Surg Technol Int.

[26] Wassenberg D, Zarmpis N, Seip N, Ambe PC (2014). Closure of small and medium size umbilical hernias with the proceed ventral patch in obese patients: a single center experience. SpringerPlus.

[27] Christoffersen MW, Helgstrand F, Rosenberg J, Kehlet H, Strandfelt P, Bisgaard T (2015). Long-term recurrence and chronic pain after repair for small umbilical or epigastric hernias: a regional cohort study. Am J Surg.

